# GPCRs as key regulators in wound healing

**DOI:** 10.3389/fcell.2026.1791888

**Published:** 2026-04-10

**Authors:** Haidi Chen, Kun Zheng, Yue Xiao, Xun Feng, Chang Zhang, Ting Zhang, Feng Luo, Huangyu Chuan, Huaping Zheng, Cheng Deng, Wei Li

**Affiliations:** 1 Center for High Altitude Medicine, Institute of High-Altitude Medicine, High Altitude Medicine Key Laboratory of Sichuan Province, Department of Dermatology and Venerology, National Clinical Research Center for Geriatrics, West China Hospital, Sichuan University, Chengdu, China; 2 Department of Dermatology and Venerology, Rare Diseases Center, West China Hospital, Sichuan University, Chengdu, Sichuan, China; 3 Department of General Dentistry, West China School of Stomatology, Sichuan University, Renmin Nanlu, Chengdu, China; 4 Harmful Components and Tar Reduction in Tobacco Key Laboratory of Sichuan Province, Chengdu, China

**Keywords:** cell proliferation, GPCR, inflammation, tissue repair, wound healing

## Abstract

Wound healing is a complex tissue repair process that occurs through a series of overlapping phases, regulated by various cell types and corresponding signaling molecules, including G-protein-coupled receptors (GPCRs). GPCRs are a large family of cell surface receptors that respond to a variety of external signals and significant targets in the design of novel drugs for a wide range of human diseases. They play vital roles throughout the different stages of wound healing. Specifically, GPCRs are essential for the recruitment of immune cells and the activation of signaling cascades related to epidermal cell proliferation and differentiation, including Hedgehog-GLI, Hippo-YAP1, and Wnt/β-catenin pathways. Modifying GPCR activity through agonists or antagonists can alter GPCR signaling pathways, potentially affecting immune cell infiltration, the production of inflammatory mediators, and wound healing rates. This review summarizes the fundamental mechanisms of GPCR signaling pathways in wound healing and highlights recent discoveries regarding the roles and functions of GPCRs in this process.

## 1 Introduction

The wound repair process is rapidly initiated following injury, facilitating the restoration of damaged tissue through a series of coordinated biological actions that comprise the tissue healing response. This process can be divided into four stages based on chronological order and the involvement of cytokines: hemostasis, inflammation, proliferation, and remodeling (Guo and Dipietro, 2010). Immediately after injury, blood vessels constrict and form a clot at the wound site to prevent further blood loss (Scully et al., 2020; Wang et al., 2025). Platelets play a crucial role by recruiting and activating immune cells, such as resident keratinocytes and fibroblasts, either through direct capture or degranulation (de Oliveira et al., 2016). Neutrophils are the first immune cells recruited from damaged blood vessels to the wound site, drawn by bacterial endotoxins, interleukin-1 (IL-1), tumor necrosis factor-α (TNF-α), and other inflammatory mediators produced by injured cells (Das et al., 2015; Komi et al., 2020; Yang et al., 2021). Once activated, neutrophils eliminate necrotic tissue and pathogens via phagocytosis and release reactive oxygen species (ROS), antimicrobial peptides, eicosanoids, and proteolytic enzymes (Segel et al., 2011). Subsequently, macrophages infiltrate the wound site, playing a pivotal role in regulating both the inflammatory response and tissue cell activity. Additionally, mast cells at the wound site become activated, releasing a variety of mediators (e.g., serotonin, histamine, LTB4, IL-1, IL-3) that promote the migration and activation of other immune cells (Wilkinson and Hardman, 2020). During the proliferation stage, hair follicle stem cells, keratinocytes, and fibroblasts are activated, leading to cell proliferation, tissue regeneration, and cytokine release. The remodeling of the extracellular matrix spans the entire process from the deposition of fibrin clots to the formation of mature collagen fibers, primarily facilitated by fibroblasts through the secretion of hyaluronic acid and proteoglycans (Darby et al., 2014).

**FIGURE 1:**
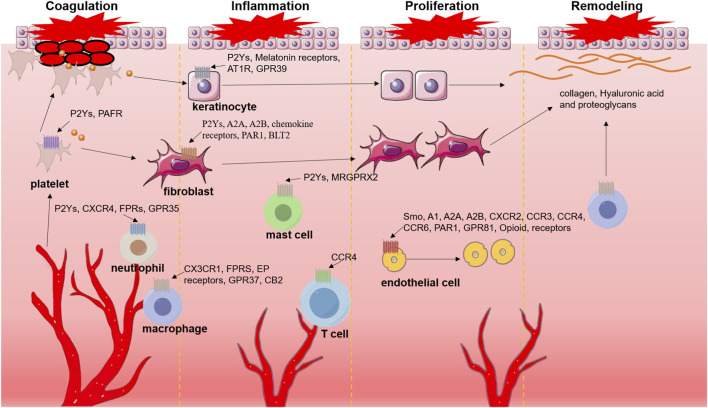
Cells involved in regulating various stages of wound healing and their expressed GPCRs. After injury, platelets rapidly accumulate at the wound site to stop bleeding and release cytokines that activate fibroblasts. In the inflammatory phase of wound healing, the cells involved are mainly immune cells, including neutrophils, macrophages, T cells, mast cells, etc. In the proliferation stage, epidermis, fibroblasts, endothelial cells and other tissue cells proliferate and differentiate. Hyaluronic acid and proteoglycans secreted by fibroblasts and various collagens expressed by macrophages and keratinocytes promote remodeling of the extracellular matrix.

G-protein-coupled receptors (GPCRs) are a class of seven-transmembrane receptors extensively expressed on the surfaces of various human cell membranes, playing a crucial role in cell signal transduction (Weis and Kobilka, 2018). The human GPCR family comprises over 800 members, categorized into six classes, each controlling diverse physiological processes (Wootten et al., 2018). Numerous studies have demonstrated that GPCRs significantly regulate cell proliferation, differentiation, and migration, including the migration of tissue and immune cells to wound sites (Kobayashi et al., 2010; Lämmermann and Kastenmüller, 2019). In the context of wound healing, distinct GPCRs regulate vasoconstriction, platelet aggregation, immune cell infiltration, and tissue cell proliferation and differentiation (Nisar et al., 2015; Pedro et al., 2020; Zmajkovicova et al., 2020) (Figure 1). Specifically, the signals transmitted by P2Y12 and platelet-activating factor receptors (PAFR) activate platelets and facilitate wound repair (Cattaneo et al., 1994; Birkl et al., 2019). Chemokine receptors, formyl peptide receptors (FPRs), and purinergic receptors are examples of GPCRs that mediate immune cell migration and regulate the inflammatory response in wounds. Additionally, GPCRs such as Smoothened (Smo), adenosine receptors, and protease-activated receptors (PARs) play roles in regulating the proliferation, differentiation, and migration of endothelial cells, macrophages, and keratinocytes (Liu et al., 2014; Bünemann et al., 2018; Hartmannsberger et al., 2024).

Here, we summarize several signaling pathways regulated by GPCRs that play pivotal roles in wound healing. Additionally, we highlight specific GPCRs that present potential targets for the development of novel therapeutic interventions in wound healing.

## 2 GPCR-regulated signaling in wound healing

G-protein-coupled receptors (GPCRs) interact with various signaling molecules—such as photons, small molecule compounds, peptides, glycoproteins, cytokines, and free fatty acids—through their extracellular domains. This interaction induces conformational changes in the receptors, enabling signal transmission into the cells (Katritch et al., 2013). The intracellular domains of GPCRs couple with heterotrimeric G proteins (Gαβγ). Upon ligand binding, the Gα and Gβγ subunits dissociate and transmit distinct downstream signals (Cabrera-Vera et al., 2003). Based on sequence homology, Gα subunits are categorized into four subclasses: Gαs, Gα (i/o), Gα (q/11), and Gα (12/13), each transmitting different downstream signals. For instance, Gq/11 induces intracellular calcium signaling, while Gs and Gi regulate cyclic adenosine monophosphate (cAMP) signaling (Gilman, 1987).

GPCRs primarily regulate the wound healing process by activating related signaling pathways (Ma S. et al., 2019; Zhang et al., 2020; Brewer et al., 2021; McEwan et al., 2021), including Hedgehog, purinergic, Hippo, and Wnt signaling. These pathways are crucial for tissue stem cell regeneration and immune cell migration.

### 2.1 Purinergic signaling in wound healing

**FIGURE 2:**
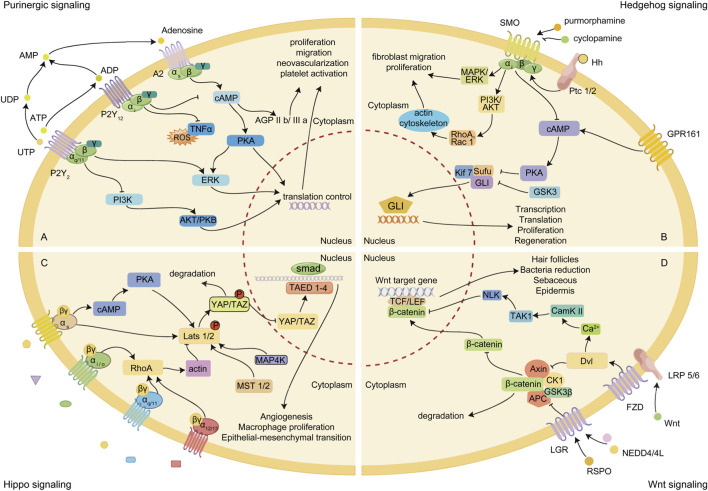
Signaling pathways mainly regulated by GPCRs in wound healing. **(A)** The purinergic signaling pathway affects wound healing by regulating platelet activation, cell proliferation and angiogenesis. **(B)** Hedgehog signaling affects wound healing in a GLI transcription factor-dependent or -independent manner. **(C)** A large number of GPCRs affect wound healing by activating the Hippo signaling pathway. **(D)** Both canonical and noncanonical Wnt signaling pathways affect wound healing.

Purinergic signaling, activated by nucleosides or nucleotides binding to purinergic receptors, plays a crucial role in regulating cell migration, proliferation, and angiogenesis (Ahmad et al., 2006; Burnstock et al., 2010; Van der Vliet and Bove, 2011) (Figure 2A). Purinergic receptors are categorized into two classes: P1 (adenosine receptors: A1, A2A, A2B, and A3) and P2 (P2Ys and P2Xs) (Von Kügelgen, 2019). Binding of ATP/UTP to the P2Y2 nucleotide receptor inhibits keratinocyte proliferation and induces lamellipodium withdrawal, disassembly of the actin network, and loss of α3 integrin expression at the cell periphery, leading to dissolution of local contacts (Taboubi et al., 2007). Additionally, activation of the P2Y2 receptor prevents growth factor-induced phosphorylation of ERK1/2 and Akt/PKB, thereby inhibiting keratinocyte spreading and migration (Haase et al., 2003; Pankow et al., 2006). These effects are dependent on the activation of Gα (q/11), with combined deletion of Gα11 and Gαq resulting in a significant migratory defect in keratinocytes, delaying cutaneous wound healing *in vivo* due to attenuated re-epithelialization (Doçi et al., 2017).

The P2Y12 receptor, activated by ADP, accelerates cutaneous wound healing, enhances new tissue formation, increases collagen deposition, and stimulates transforming growth factor-β (TGF-β) production in the wound (Cattaneo, 2015). Binding of ADP to P2Y12 elevates levels of reactive oxygen species (ROS) and interleukin-13 (IL-13) while reducing tumor necrosis factor-α (TNF-α) levels, leading to an increase in inflammatory cells such as neutrophils, eosinophils, mast cells, and gamma delta (γδ) T cells, while decreasing regulatory T (Treg) cells in the lesion (Gachet, 2006). Consequently, ADP enhances fibroblast proliferation and migration, myofibroblast differentiation, and keratinocyte proliferation in a P2Y12-dependent manner. ADP functions as a pro-resolution mediator in diabetes-associated skin wounds and is a promising target for therapeutic intervention in this widespread issue (Borges et al., 2021).

Activation of adenosine A1, A2A, and A2B receptors promotes the proliferation of human endothelial cells, accelerating wound healing (Bonyanian et al., 2019). The migration of fibroblasts and endothelial cells, as well as angiogenesis, is also enhanced by the activation of A2A and A2B receptors (Montesinos et al., 1997; Macedo et al., 2007). Adenosine, a vital regulator of neovascularization, promotes angiogenesis by inducing the proliferation and migration of endothelial cells, tube formation, and the homing of endothelial progenitor cells to the site of tissue injury (Grant et al., 2001; Dubey et al., 2002; Montesinos et al., 2004). Beyond these canonical pathways, emerging research has unveiled a novel and potent mechanism mediated by the adenosine A2A receptor (A2AR). Notably, the activation of A2AR has been found to initiate a signaling cascade culminating in the stabilization and nuclear translocation of the transcriptional coactivator Yes-associated protein (YAP). This A2AR-YAP axis serves as a critical regulator of gene programs essential for cell proliferation, migration, and tissue remodeling during repair. This mechanism has been particularly elucidated in the context of corneal wound healing, where it coordinates a robust regenerative response (Sun et al., 2024).

### 2.2 Hedgehog signaling in wound healing

Hedgehog (Hh) signaling, mediated by the G protein-coupled receptor Smoothened (Smo), activating Smo-coupled Gα (i/o) proteins, including Gα (i1), Gα (i2), Gα (i3), and Gα (o)plays a crucial role in cell fate decisions during embryogenesis and contributes significantly to tissue homeostasis and wound healing postnatally (Asai et al., 2006; Hall et al., 2019) (Figure 2B).

Notably, the agonist purmorphamine and the antagonist cyclopamine can directly target Smo to regulate the Hh signaling pathway (Taipale et al., 2000; Sinha and Chen, 2006; Barzi et al., 2011). The execution of Gα (i/o) signaling relies on the downregulation of cAMP, with GPCRs such as GPR161, which upregulate cAMP levels, inhibiting Shh signaling (Mukhopadhyay et al., 2013; He and Lu, 2015). Research indicates that both Shh and Smo agonists, such as purmorphamine, stimulate cell migration, while the Smo inhibitor KAAD-cyclopamine abolishes this increased motility (Polizio et al., 2011). Activation of the Shh pathway enhances dermal regeneration, facilitates hair follicle neogenesis (HFN), and reduces scarring at the wound site (Lim et al., 2018). Additionally, Shh signaling has been shown to promote dermal papilla formation in scarring wounds, a process partially dependent on Wnt signaling. Moreover, neuro-epithelial interactions of stem cells in mouse hair follicles and sensory-tactile dome epithelium are regulated by Shh and Smo (Xiao et al., 2015).

Beyond the canonical Hh signaling pathway that activates Gli transcription factors, noncanonical pathways independent of PKA and Gli transcriptional activity also exist (Wei et al., 2025). Smo activation can mediate the proliferation and migration of rheumatoid arthritis fibroblast-like synoviocytes (RA-FLSs) via the MAPK/ERK pathway, suggesting a similar mechanism may be present in wound healing-related cells (Elia et al., 2007; Liu et al., 2018).

### 2.3 Hippo signaling in wound healing

Hippo signaling plays a crucial role in regulating organ size, epithelial homeostasis, tissue regeneration, and immune modulation (Huang et al., 2025). In the context of wound healing, this pathway is integral to the differentiation of fibroblasts and endothelial cells, as well as subsequent tissue regeneration (Brewer et al., 2021; Yang et al., 2023). Yes-associated protein (YAP) and transcriptional coactivator with PDZ-binding motif (TAZ) are two key downstream transcription coactivators in this pathway, which mediate the major gene regulation and biological functions of the Hippo pathway (Ma T. et al., 2019) (Figure 2C).

The Hippo pathway significantly influences cell migration and proliferation, processes tightly regulated by GPCRs (Ehmer and Sage, 2016). Activation of GPCRs that couple to G12/13, Gq/11, or Gi/o, downregulates YAP/TAZ phosphorylation. Conversely, GPCRs coupled to Gs, such as adrenergic receptor β2, increase YAP/TAZ phosphorylation (Yu et al., 2012). The stability of YAP and TAZ is vital for accelerating wound healing. Deletion of YAP and TAZ markedly delays wound closure and reduces TGF-β1 expression at wound sites. TEAD, the key downstream transcription factor of YAP/TAZ, is essential for promoting cell proliferation and inducing epithelial-mesenchymal transition (Lee M. J. et al., 2014). Disruption of TEAD-TAZ binding or silencing TEAD expression inhibits TAZ’s ability to promote these processes. Thus, the Hippo signaling pathway, regulated by various GPCRs, plays a fundamental role in wound healing by modulating the activity and stability of YAP and TAZ (Zhang et al., 2009).

### 2.4 Wnt signaling in wound healing

The Wnt signaling pathway is critically involved in determining somatic stem cell fate, self-renewal, and differentiation, and plays a significant role in anti-inflammation and cell proliferation during wound healing. Activation of Wnt/β-catenin signaling enhances the functions and numbers of endothelial progenitor cells, thereby promoting angiogenesis and vasculogenesis. Concurrently, Wnt signaling activation stimulates epidermal stem cell proliferation, keratinocyte differentiation and migration, and hair follicle regeneration, culminating in enhanced wound healing.

In the Wnt pathway, extracellular signals are transmitted into cells primarily through frizzled GPCR (FZD), lipoprotein receptor-related protein (LRP), and receptor tyrosine kinase-like orphan receptor (ROR) (Oh et al., 2024). FZDs serve as the main Wnt receptors and can transduce signals either independently or in complex with LRP5 or LRP6. Single-cell transcriptomic data show that both Lgr5^+^ and Lgr6^+^ cells rapidly induce a genetic wound signature after injury, such as the remodeling of receptors, to allow them to interact with the wound environment (Joost et al., 2018). The Wnt signaling pathway is categorized into canonical and noncanonical pathways based on the involvement of β-catenin. In the canonical pathway, β-catenin shuttles between the cytoplasm and nucleus, playing roles in adhesion and transcription regulation. In the noncanonical Wnt pathway, Wnt binding to FZD receptors influences intracellular calcium signaling and negatively regulates the canonical Wnt pathway.

Wnt/β-catenin signaling is essential for hair follicle development (Huelsken et al., 2001). Skin-wide deletion of β-catenin leads to stem cell depletion and hair loss, while continuous activation of Wnt in the skin leads to initial proliferation of hair follicles followed by depletion of stem cells and premature hair loss (Choi et al., 2013).

## 3 GPCRs regulate various stages of wound healing

### 3.1 GPCRs regulate the inflammatory stage of wound healing

After injury, resident immune cells are activated by damage-associated molecules released by necrotic cells and pathogen lysis products (Martin and Leibovich, 2005). Many GPCRs are expressed in inflammation-related immune cells and play crucial roles in their activation and recruitment to inflammatory sites (Martins-Green et al., 2013) (Table 1). Although inflammation serves to prevent infection by external pathogens, it can also impair normal physiological functions and delay wound healing. The resolution phase of inflammation is tightly regulated by anti-inflammatory and pro-repair mediators, many of which target GPCRs to promote inflammation resolution (Im, 2020; Quiros, 2020).

**TABLE 1:** Inflammation-related GPCRs in wound healing.

Receptor	Ligands	Function	Ref.
CXCR2	Ac-PGP	Mediating endothelials cell migration and proliferation	Kwon et al. (2017)
CXCR4	CKCL12	Mediating neutrophils migration	Furze and Rankin (2008); Wang et al. (2017)
CX3CR1	CX3CL1	Mediating monocytes/macrophages recruitment	Ishida et al. (2008)
CCR4	CCL17, CCL22	Activation and recruitment of regulatory T cells	Iellem et al. (2001); Kato et al. (2011)
CCR10	CCL28	Inhibiting angiogenesis	Chen et al. (2020)
CCR2	CCL2	Inducing monocytes accumulation	Raghu et al. (2017); Boniakowski et al. (2018); Kuang et al. (2021)
FPRs	N-formyl peptides	Mediating neutrophils infiltration	Liu et al. (2014); Leslie et al. (2020)
EP receptors	PEG 2	Regulating macrophages proliferation	Zhang et al. (2018); Gilman and Limesand (2021)
GPR35	5-HIAA	Mediating neutrophils activation and migration	De Giovanni et al. (2022)
GPR37	NPD1 prosaptide TX14	Activating macrophages	Bang et al. (2018)

Chemotaxis GPCRs, which mediate leukocyte chemotaxis, are pivotal in regulating innate and adaptive immune responses, thereby influencing wound healing processes. CC and CXC chemokine receptors, widely expressed in the epidermis (CCR3, CCR4, CCR6, CXCR1, CXCR3), dermal fibroblasts (CCR3, CCR4, CCR10), and microvascular endothelial cells (CCR3, CCR4, CCR6, CCR8, CCR9, CCR10, CXCR1, CXCR2, CXCR3), significantly enhance wound repair upon activation (Bünemann et al., 2018). For instance, CXCR2 in human endothelial progenitor cells (hEPCs) promotes migration, proliferation, and neovascularization in response to glycine-proline (Ac-PGP), which benefits cutaneous dermal wounds (Kwon et al., 2017). CXCR4 is closely associated with tissue regeneration, participating in wound healing by regulating multiple signaling pathways to promote stem cell survival and proliferation (Chen et al., 2021). The CXCL12/CXCR4 signaling axis also mediates the migration of aged and injury-derived neutrophils back to the bone marrow for apoptosis (Furze and Rankin, 2008; Wang et al., 2017). Additionally, CX3CR1/CX3CL1 facilitates the recruitment of bone marrow-derived monocytes/macrophages, which release profibrotic and angiogenic mediators, such as TGF-β1 and vascular endothelial growth factor, aiding skin wound repair (Ishida et al., 2008). CCR4 activation by its ligands CCL17 and CCL22 impedes diabetic wound healing by recruiting and activating regulatory T cells at inflammation sites (Iellem et al., 2001; Kato et al., 2011; Jonczyk et al., 2024). Conversely, CCR4 depletion, which prevents the recruitment of regulatory T cells to the wound site, accelerates wound healing in diabetic mice (Barros et al., 2019). CCL28/CCR10 signaling inhibits skin wound angiogenesis in an endothelial nitric oxide synthase (eNOS)-dependent manner, involving interactions with Src, PI3K, and MAPK signaling. A myristoylated peptide that blocks CCR10-eNOS interaction promotes eNOS activity and upregulates nitric oxide levels, thereby enhancing angiogenesis and wound healing in mice (Chen et al., 2020). CCR2, overexpressed in monocytes, induces their accumulation at wound sites, mediating the generation and resolution of inflammation, even in diabetic wound healing (Raghu et al., 2017; Boniakowski et al., 2018; Kuang et al., 2021).

The G protein-coupled formyl-peptide receptors (FPRs), including FPR1, FPR2, and FPR3, couple with Gi protein to regulate downstream signaling (Rabiet et al., 2011; Li et al., 2016). FPR1 and FPR2, activated by N-formyl peptides from bacterial or host cell mitochondrial protein degradation during inflammation, are primarily expressed in leukocytes such as neutrophils, monocytes, macrophages, natural killer cells, and dendritic cells, mediating their recruitment to inflammation sites (Le et al., 2001; Yang et al., 2002; Kim et al., 2009). Neutrophil infiltration in inflammation and injury, mediated by pathogen-derived and host-derived chemotactic ligands binding to FPRs, promotes wound healing (Liu et al., 2014; Leslie et al., 2020). However, FPR-mediated chemotaxis signaling in neutrophils can be impaired in diabetic wounds, reducing cell chemotaxis and making these wounds more susceptible to infection (Roy et al., 2022). WD40 repeat protein (WDR)-26 can bind to FPR1, inhibiting the activation of Rac family small GTPase 1, the cell division cycle 42, and ROS production, thereby delaying intestinal epithelial wound healing. Administration of N-formyl-methionyl-leucyl-phenylalanine can dissociate WDR26 from FPR1, abolishing this inhibition (Hasegawa et al., 2020).

Prostanoid E (EP) receptors, including EP1, EP2, EP3, and EP4, are GPCRs that couple with different G proteins: EP1 with Gq, EP3 with Gi, and EP2 and EP4 with Gs, activating distinct downstream signaling pathways that lead to pro- or anti-inflammatory responses (Ricciotti and FitzGerald, 2011). Prostaglandin E2 (PGE2) interaction with EP receptors is crucial for an appropriate inflammatory response, influencing macrophage proliferation in a dose-dependent manner. PGE2 treatment reduces M1 macrophages while increasing M2 macrophages and related products such as arginase, IL-1α, IL-10, CD68, and CD206, while decreasing IL-1β, IL-6, and TNF-α (Zhang et al., 2018). Hydrogels incorporating PGE2 have therapeutic effects on skin lesions in mice, increasing macrophages, promoting angiogenesis, and allowing wounds to close faster and with less scarring (Gilman and Limesand, 2021).

GPR35 mediates neutrophil activation and promotes their migration from blood vessels into inflamed tissue in response to the serotonin metabolite 5-HIAA in mice (De Giovanni et al., 2022). In mucosal repair, GPR35 signaling promotes wound healing by inducing fibronectin and integrin α5 expression, coupling to Gi protein, and activating ERK1/2 in colonic epithelial cells (Tsukahara et al., 2017). GPR37, expressed by macrophages, contributes to the resolution of inflammatory pain. Activation of GPR37 by neuroprotectin D1 (NPD1) and the prosaptide TX14 triggers macrophage phagocytosis of zymosan particles and promotes the resolution of inflammatory pain via calcium signaling (Bang et al., 2018).

Overall, GPCRs play critical roles in the activation, recruitment, and regulation of inflammation-related immune cells during wound healing. Their modulation offers significant therapeutic potential for enhancing tissue regeneration and repair.

### 3.2 GPCRs regulate the proliferative stage of wound healing

In the proliferative phase of wound healing, keratinocytes, endothelial cells, and fibroblasts are rapidly activated and proliferate, promoting the formation of new tissues. Numerous studies have demonstrated that various GPCRs regulate the proliferation of these cells (Pedro et al., 2020) (Table 2).

**TABLE 2:** Proliferation-related GPCRs involved in wound healing.

Receptor	Ligand	Cell type	Function	Ref.
PARs	PARs-activating peptides	fibroblasts and endothelial	Promoting cells viability and migration	Seo et al. (2021); Bandara and MacNaughton (2022)
GPR81	lactate	vascular progenitor cells and endothelial cells	Promoting cell migration and angiogenesis	Farooq et al. (2014); Hoque et al. (2014)
GPR120	DHA	endothelial cells	Inhibiting VEGF-induced cell migration	Chao et al. (2014)
TGR5	deoxycholic acid	intestinal epithelial cells	Inhibiting wound healing	Duboc et al. (2014); Azuma et al. (2022)
PAFR	platelet-activating factor	epithelial cells	Promoting intestinal mucosal repair	Birkl et al. (2019)
opioid receptors	β-endorphin morphine homologues	endothelial cells	Promoting cell proliferation	Wang et al. (2017)
melatonin receptor	melatonin	keratinocytes	Promoting keratinocyte proliferation	Lee S. J. et al. (2014); Slominski et al. (2018)
AT1R	angiotensin II	keratinocytes and fibroblasts	Inducing cell migration	Yahata et al. (2006)
GPR39	zinc	fibroblasts and keratinocytes	Promoting skin development and homeostasis	Zhao et al. (2015); Satianrapapong et al. (2020)

PARs are a distinct class of GPCRs activated by protease cleavage of their extracellular domain (Adams et al., 2011; Li et al., 2024). Activation of PAR1 by thrombin and PAR1-activating peptide (AP) enhances fibroblast and endothelial cell viability and migration, thereby significantly promoting skin wound healing by upregulating the gene expression of TGF-β, fibronectin, and type I collagen (Hamilton and Trejo, 2017; Seo et al., 2021). PAR2 contributes to intestinal wound resolution through COX-2 overexpression and EGFR transactivation and promotes bronchial epithelial cell (BEC) migration and proliferation after cleavage by tryptase, facilitating epithelial wound healing (Mogren et al., 2021; Bandara and MacNaughton, 2022). GPR81 activation by lactic acid enhances endothelial cell migration and blood vessel formation by upregulating β-arrestin expression, independent of the cAMP/PKA pathway (Farooq et al., 2014; Hoque et al., 2014). *In vivo*, lactate administration reduces inflammation and organ injury in mice with immune hepatitis via a GPR81-dependent mechanism. GPR120, also known as free fatty acid receptor 4 (FFA4), is activated by docosahexaenoic acid (DHA) binding, which inhibits VEGF-induced cell migration (Chao et al., 2014). GPR120 is also implicated in ω-3 fatty acid-induced proliferation of bronchiole epithelial cells in mice (Lee et al., 2017).

TGR5, expressed in small intestinal epithelial cells, recognizes various bile acids as ligands (Duboc et al., 2014). Deoxycholic acid (DCA) binding to TGR5 inhibits wound healing through AKT signaling activation (Azuma et al., 2022). Platelet-activating factor receptor (PAFR) activation by platelet-activating factor (PAF) promotes wound closure via interactions with the EGFR, Src, and Rac1 signaling pathways (Birkl et al., 2019). In epithelial cells, TNF-α promotes intestinal mucosal repair by upregulating PAFR expression. Opioid receptors in endothelial cells mediate signals from morphine and its homologs, promoting cell proliferation via Opioid peptide β-endorphin (β-END) produced by keratinocytes (Wang et al., 2017).

Melatonin receptor activation by melatonin induces the expression of involucrin, keratin-10, and keratin-14, markers of keratinocyte proliferation and differentiation (Slominski et al., 2018). Melatonin increases the proliferative activity of keratinocytes *in vitro* and activates the downstream PKC signaling pathway via Gαq coupling, promoting skin wound healing in mice (Lee et al., 2014). Activation of the Ang II type 1 receptor (AT1R) by angiotensin II (Ang II) induces EGFR transactivation dependent on HB-EGF shedding, promoting keratinocyte and fibroblast migration to wound sites (Yahata et al., 2006).

GPR39, a member of the ghrelin family of GPCRs, is activated by zinc and regulates neuronal excitability, vascular tone, and immune response homeostasis. During skin wound repair, GPR39 is spatiotemporally expressed in the sebaceous gland (SG), promoting skin development and homeostasis (Sato et al., 2016; Xu et al., 2021). GPR39 regulates keratinocyte proliferation via a PI3K-MKK-ERK-dependent mechanism. Additionally, GPR39 agonists, such as TC-G 1008, enhance keratinocyte proliferation and ERK phosphorylation in a time- and concentration-dependent manner, thereby benefiting cutaneous wound treatment (Zhao et al., 2015; Satianrapapong et al., 2020).

In summary, GPCRs play critical roles in the proliferative phase of wound healing by regulating the proliferation and migration of key cell types, including keratinocytes, endothelial cells, and fibroblasts. Their activation and signaling pathways significantly contribute to tissue formation and wound repair.

### 3.3 GPCRs regulate the remodeling stage of wound healing

Re-epithelialization in wound healing involves the infiltration of dermal cells and deposition of the extracellular matrix, primarily regulated by growth factors such as transforming growth factors and adhesion molecules (Reynolds et al., 2005). This process is also modulated by several proliferation- and differentiation-related G protein-coupled receptors (GPCRs) (Table 3).

**TABLE 3:** The re-epithelization-related GPCRs in wound healing.

Receptors	Ligands	Cell type	Function	Ref.
β2-AR	adrenaline	keratinocytes	Inhibiting keratinocyte migration, and re-epithelialization	Luqman and Götz, (2021)
BLT2	LTB4, CAY10583	keratinocytes, fibroblasts	Enhancing keratinocyte migration and indirect stimulation of fibroblast activity	Luo et al. (2017), Matsumoto et al. (2020)
GPR124	Unknown	endothelial cells	Promoting cell migration and adhesion	Hernández-Vásquez et al. (2017)
CB2	Gp1a	keratinocytes	Promoting keratinocyte proliferation and migration	Wang et al. (2016); Zhao et al. (2021)
CaSR	Ca^2+^	keratinocytes	Inducing cell adhesion and differentiation	Tu et al. (2019)

β-adrenergic receptors (β-ARs) are crucial functional regulators in various physiological systems, including vascular system, endocrine system, and central nervous system, and they play a significant role in wound healing (Pullar et al., 2006). In response to stress, injured epithelial cells release epinephrine (adrenaline), which activates β2-AR, impairing keratinocyte migration and re-epithelialization (Luqman and Götz, 2021). β2-AR activation enhances the transcription of the hyaluronan synthase 2 (HAS2) gene via the Gs-adenylyl cyclase-PKA-CREB signal transduction pathway, increasing hyaluronan production. This plays a vital role in inflammatory reactions and tissue repair by maintaining tissue elasticity and physical properties (Heldin et al., 2019; Kuroda and Higashi, 2021).

Leukotriene B4 receptor type 2 (LTB4R2) activation accelerates wound healing, particularly for intractable wounds such as diabetic ulcers (Liu et al., 2014). The agonist CAY10583 enhances wound healing by promoting keratinocyte migration and indirectly stimulating fibroblast activity in diabetic rats through Gi/o protein-dependent and PLC/PKC signaling pathways (Luo et al., 2017; Matsumoto et al., 2020).

GPR124 contributes to polarity acquisition is essential for the directional migration of cells towards the wound site, ensuring effective angiogenesis and tissue regeneration. GPR124 interacts with Rho guanine nucleotide exchange factors, such as Elmo and Dock, which are involved in the activation of Rho GTPases. This interaction is important for cytoskeletal reorganization, cell migration, and cell adhesion (Hernández-Vásquez et al., 2017).

Cannabinoid receptor 2 (CB2) activation by Gp1α reduces inflammation and fibrogenesis while promoting wound healing and re-epithelialization by inhibiting neutrophil and macrophage infiltration and increasing keratinocyte proliferation and migration (Zhao et al., 2021; Wang et al., 2016). The deficiency of cannabinoid receptor 1 (CB1) leads to excessive expression of monocyte chemoattractant protein (MCP)-1 and TNF-α in the early stages of wound healing, showed CB1 is essential for wound healing process (Ruhl et al., 2021).

The Ca^2+^-sensing receptor (CaSR) is expressed in keratinocytes, where it senses changes in extracellular calcium levels. This sensing mechanism is crucial for keratinocyte proliferation and migration, two key processes in re-epithelialization during wound healing (Tu and Bikle, 2013; Tu et al., 2019). CaSR also enhances cell adhesion in keratinocytes by regulating the expression and function of adhesion molecules such as E-cadherin, which stabilizes the newly formed epithelial layer and prevents wound contraction. Improved cell adhesion facilitates the integrity and cohesion of the epithelial layer, promoting efficient wound closure (An et al., 2022).

In summary, the re-epithelialization phase of wound healing is a complex process regulated by multiple GPCRs that influence cell proliferation, migration, adhesion, and differentiation. These receptors play crucial roles in coordinating the cellular and molecular mechanisms required for effective wound repair.

### 3.4 Other wound healing-related GPCRs

Endothelin-1 (ET-1) receptors, ETA and ETB, play distinct roles in the wound healing process. ETA receptor activation drives vasoconstriction, hemostasis, and the initiation of inflammation, which are critical for early wound repair. ETA also inhibits smooth muscle cell (SMC) proliferation, potentially limiting scar formation. Conversely, the ETB receptor is involved in clearing ET-1, mediating vasodilation, and stimulating angiogenesis through the release of nitric oxide and prostacyclin, thereby enhancing tissue perfusion and neovascularization. In SMCs, ETB promotes proliferation and migration, contributing to granulation tissue formation and wound closure. Additionally, ETB exerts anti-inflammatory and pro-fibrinolytic effects, aiding in the resolution of inflammation and matrix remodeling during the later stages of wound healing (Granchi et al., 2002; Houde et al., 2016).

Histamine receptors H1 (H1R) and H2 (H2R) also play crucial roles in various physiological responses (Rosenbaum et al., 2009). H1R, predominantly coupled to Gq proteins, is expressed on smooth muscle cells, endothelial cells, and neurons. Activation of H1R regulates intracellular calcium mobilization, eliciting responses such as bronchoconstriction, intestinal cramping due to smooth muscle contraction, edema from increased vascular permeability, and itching and pain from the stimulation of sensory nerve endings (Simons, 2004). Conversely, H2R primarily regulates gastric acid secretion and influences gastrointestinal motility and intestinal secretion (Del Valle and Gantz, 1997). H2R antagonists inhibit the cAMP signaling pathway by competitively binding with histamine, thereby reducing gastric acid secretion. The therapeutic efficacy of H2R antagonists in promoting gastric and duodenal ulcer healing is dependent on the degree and duration of gastric acid inhibition during therapy (Deakin and Williams, 1992).

## 4 Medications target GPCRs in wound healing

### 4.1 Prostacyclin analog

Alprostadil, a synthetic analog of PGE1, can reduce anastomotic leaks, strengthen anastomoses, and accelerate wound healing in rat models by modulating Wnt1 and β-catenin mRNA expression (Zheng et al., 2018). Clinically, alprostadil has been beneficial for ischemic lower limb ulcers, improving ulcer size, pain, and oxygen pressure when administered subcutaneously, and achieving complete healing in refractory Sneddon syndrome-related skin ulcers upon intravenous administration.

Beraprost sodium (BPS), a synthetic PGE1 analogue, targets the Prostacyclin I2 (PGI2) receptor produced by cyclooxygenase-2 (COX-2). BPS induces smooth muscle relaxation and vasodilation via binding with PGI2, shown efficacy in accelerating wound healing in chronic diabetic foot ulcers. Clinical studies revealed that oral administration of BPS significantly improved median healing rates (88.1% vs. 33.3%) and complete healing rates (48% vs. 8%) compared to controls (Awsakulsutthi et al., 2014).

Iloprost, a synthetic prostacyclin analog, enhances wound healing processes, particularly in patients with chronic or critical limb ischemia. Iloprost exerts vasodilation, inhibits platelet aggregation, and provides anti-inflammatory effects, improving perfusion and microcirculation at the wound site (Legrand et al., 2025). Clinical evidence shows that Iloprost accelerates the healing of ischemic wounds, reduces pain, and increases walking distance in patients with peripheral arterial disease, ultimately reducing the risk of major amputations (Tello et al., 2022). It is particularly effective in augmenting the healing of chronic diabetic foot ulcers, both as an adjunct to drug-eluting balloon angioplasty and in standalone treatments.

Treprostinil has shown promise in promoting wound healing, particularly for critical limb ischemia (CLI) and systemic sclerosis (SSc)-related ulcers. In a pilot study involving CLI patients, continuous subcutaneous treprostinil therapy led to significant reductions in ischemic rest pain and facilitated complete wound healing in several subjects over a 12-week period (Berman et al., 2006). In an experimental murine model, treprostinil iontophoresis significantly improved the healing of excisional wounds, increased microvascular density, and reduced inflammation, specifically in urokinase-type plasminogen activator receptor (uPAR)-deficient mice. This effect was not observed in HOCl-treated mice or with electrostimulation alone, indicating treprostinil’s specific efficacy in enhancing wound healing under conditions mimicking SSc-related ulcers (Kotzki et al., 2022).

### 4.2 Histamine receptor antagonist

Diphenhydramine, a first-generation histamine H1 receptor antagonist. Its role in wound healing has been investigated, primarily focusing on its anti-inflammatory properties and its ability to reduce itch and pain. Diphenhydramine effectively reduces inflammation and itch in patients with allergic dermatitis, which is beneficial for secondary wound healing, and accelerate wound healing by promoting re-epithelialization (Shenefelt, 2010; Marsella and De Benedetto, 2017; De Nardi et al., 2022).

Ranitidine, an H2-receptor antagonist primarily used to reduce stomach acid, has shown potential benefits in wound healing and clinical research (Strum, 1983). It appears to promote wound healing by enhancing cell proliferation, collagen synthesis, and angiogenesis, which are critical for tissue repair. Clinical research suggests that ranitidine can shorten the healing time and improve the quality of wound repair, particularly in cases where gastric issues might contribute to delayed wound healing.

### 4.3 β-AR antagonist

Timolol is a non-selective beta-adrenergic receptor blocking agent, often used in the treatment of glaucoma and hypertension, shown potential in wound healing and has been the subject of various clinical research studies. Several clinical studies have evaluated its efficacy in this context. For example, timolol has been investigated for the treatment of atrophic acne scars as well as postoperative breast scars (Hawwas et al., 2023; Nafissi et al., 2025). The results of these studies have been largely positive, with Timolol showing significant improvements in scar reduction.

### 4.4 Bosentan

Bosentan, an oral dual endothelin-1 receptor antagonist, demonstrates promising potential in promoting postoperative wound healing and resolving chronic non-healing ulcers, particularly in patients with systemic sclerosis (SSc) and Raynaud phenomenon-related complications. It effectively treats digital ulcers in SSc due to its blockade of ETA and ETB receptors, contributing to improved peripheral circulation and tissue repair. Off-label usage of bosentan accelerates healing of nondigital lower extremity ulcers, especially those exhibiting cyanosis, even in cases refractory to conventional therapies like vasodilators, prostanoids, and local interventions (Naert and De Haes, 2013; Huh et al., 2021).

### 4.5 Other candidate medications and compounds

In addtion to the aforementioned agents, several other clinically significant medications, including ramipril, losartan and ambrisentan. These agents, acting either as antagonists or agonists at specific GPCR targets, collectively contribute to vasodilation by relaxing vascular smooth muscle. This action fosters enhanced microcirculatory blood flow, thereby exerting antihypertensive effects and promoting improved perfusion to peripheral tissues (Warner and Perry, 2002; Al-Majed et al., 2015; Rivera-Lebron and Risbano, 2017). However, there appears to be a paucity of direct research specifically investigating their impact on the process of wound healing.

Beyond pharmaceutical interventions, a variety of compounds have been investigated for their potential to augment wound healing processes. PGE2 has been incorporated into chitosan (CS) hydrogels to form CS + PGE2 hydrogels, which serve as a delivery system to prolong the release of PGE2 and enhance its therapeutic effects. This hydrogel system has demonstrated improved tissue repair and regeneration capabilities in murine models of cutaneous wound healing (Futagami et al., 2002). AMD3100, a CXCR4 antagonist, demonstrates accelerated wound healing in diabetic mice by enhancing the recruitment of bone marrow-derived endothelial progenitor cells (EPCs) to the regenerating vasculature through disruption of CXCR4-SDF-1 interactions. These findings suggest that AMD3100 accelerates diabetic wound repair by modulating the chemokine milieu, stimulating angiogenesis, and orchestrating inflammatory responses crucial for tissue regeneration (Nishimura et al., 2012). These medications and compounds which targeting GPCRs suggest more potential in the treatment on the wound healing in clinic.

## 5 Conclusion and perspectives

In conclusion, GPCRs are involved in each stage of the wound healing process with distinct roles and perform different functions. They play an important role in the regulation of immune responses in the inflammatory phase and cell proliferation and differentiation in the tissue regeneration phase during damage repair. Cutaneous wound repair and regeneration are mainly related to dermal fibroblasts and epidermal stem cells. While GPCRs, P2Y12, Smo, PAR1, etc., can all influence the behavior of fibroblasts through their downstream signals. And Wnt signaling pathway regulates the proliferation and differentiation of epidermal stem cells. Numerous studies have confirmed the important role of GPCRs in diseases such as diabetes, obesity, AD, and psychiatric disorders, providing a strong impetus for drug discovery and development efforts in this field. Similarly, GPCRs affect the process of wound healing by regulating the behavior of various cells, including neutrophils, macrophages, T cells, DCs, keratinocytes, fibroblasts, endothelial cells, etc. Given the stage-specific roles of GPCRs in wound healing and the need for defined molecular mechanisms in therapeutic development, we summarize the core receptors, functions, potential drugs/compounds and signalling pathways in Table 4.

**TABLE 4:** Key GPCR targets and therapeutic opportunities in wound healing.

Receptors	Primary roles in wound healing	Possible molecular mechanisms	(Potential) drugs/Compounds	Ref.
P2Y2	Inhibits keratinocyte cell spreading and induces lamellipodium withdrawal	Prevents growth factor-induced phosphorylation of Erk (1,2) and Akt/PkB	ATP (agonist)	Taboubi et al. (2007)
P2Y12	Accelerates wound closure and improves tissue repair represented by type I collagen deposit and adequate reepithelization	Increase of neutrophils, eosinophils, mast cells, M2 macrophages, myofibroblasts and Vγ4+ and Vγ5+ cells in the wound	ADP (agonist)	Borges et al. (2021)
A2AR	Enhances angiogenesis; promotes fibroblast and endothelial cell migration; regulates inflammation via A2AR-YAP axis	Stabilizes YAP and promotes its nuclear translocation	CGS21680 (agonist)	Sun et al. (2024)
Smoothened (Smo)	Promotes fibroblast migration and angiogenic tubulogenesis via non-canonical Hedgehog signaling	Activates Smo-Gi-PI3K axis to stimulate RhoA/Rac1, inducing cytoskeletal changes independent of Gli transcription	Purmorphamine (agonist)	Polizio et al. (2011)
CXCR2	Mediates endothelial cell migration and proliferation	PI3K/AKT pathway	Ac-PGP (agonist)	Kwon et al. (2017)
CXCR4	Modulates​ the chemokine milieu, stimulates​ angiogenesis, and orchestrates​ inflammatory responses crucial for tissue regeneration	Disrupts CXCR4-SDF-1 interactions	AMD3100 (antagonist)	Nishimura et al. (2012)
CX3CR1	Mediates monocytes/macrophages recruitment	AMPK/PPARγ pathway	r-FKN (recombinant fractalkine)	Chen et al. (2023)
CCR4	Activates and promotes the recruitment of regulatory T cells	—	Mogamulizumab (monoclonal antibody)	Kim et al. (2018)
CCR10	Inhibits angiogenesis	Endothelial nitric oxide synthase (eNOS)-dependent Src, PI3K, and MAPK signaling	—	Chen et al. (2020)
CCR2	Induces monocyte accumulation	CCR2 inhibition reduces fibrosis and cholestasis in animal models of PSC	Cenicriviroc (CVC) (CCR2/5 antagonist)	Guicciardi et al. (2018)
FPRs	Mediates neutrophils infiltration	Enhances the production of TNF-α by microglia/macrophages	T0080 (antagonist)	Leslie et al. (2020); Li et al. (2024)
EP receptors	Activates Wnt/β-catenin signal pathway	Modulates​ Wnt1 and β-catenin mRNA expression	Alprostadil (agonist)	Zheng et al. (2018)
GPR35	Promotes wound healing in intestinal and diabetic models by enhancing epithelial cell migration and suppressing oxidative stress	Gi protein coupling, decreased cAMP, ERK1/2 activation, upregulation of fibronectin and integrin α5, interaction with OXSR1	YE120, Zaprinast, Pamoic acid (agonist)	Tsukahara et al. (2017); Li et al. (2024)
GPR81	Promotes cell proliferation, colony formation, and wound healing (cell migration) in breast cancer cells	Activation by lactate leads to Gi protein coupling; upregulates genes for mitochondrial biogenesis (PGC1α, SIRT1) and oxidative phosphorylation (COX IV, ATPsyn); enhances antioxidant response (HMOX1, NQO1) via IGFBP6 upregulation	Lactate, 3,5-DHBA (agonist)	Longhitano et al. (2022)
GPR120	Accelerates skin wound healing by promoting re-epithelialization, reducing inflammation, and enhancing tissue repair	Recruits β-arrestin, promotes internalization, modulates cytokines by reducing IL-1β and elevating IL-6 and TGF-β, and increases markers such as involucrin	DHA Pinocembrin (agonist)	Arantes et al. (2016), Mazzotta et al. (2021)
TGR5	Promotes colonic mucosal healing by enhancing intestinal epithelial cell migration and restitution, accelerating wound closure in models of colitis	Triggers cAMP/PKA signaling, induces IP3R-mediated Ca2+ release, promotes actin polymerization, and enhances cell migration	7-KDCA, LCA (agonist)	Azumaet al. (2022), Zhang et al. (2025)
PAFR	Promotes mucosal repair by enhancing epithelial migration	Activates Src/ADAM10-EGFR and Rac1/ROS/FAK pathways	PAF (agonist)	Birkl et al. (2019)
opioid receptors	Enhances the healing of ischemic wounds and accelerating wound closure through increased keratinocyte migration	Inhibits neuropeptides and stimulates endothelial growth via MAPK/ERK	Morphine (agonist)	Wang et al. (2017)
AT1R	Context-dependent: Promotes migration in acute phase; its blockade accelerates healing in chronic wounds	Dual mechanisms: 1) Acute: HB-EGF/EGFR transactivation. 2) Chronic blockade: Selective SMAD modulation (inhibits SMAD1/5/9; activates SMAD2/3/4)	Angiotensin II (Agonist); Losartan, Valsartan (Antagonists)	Yahata et al. (2006), Abadir et al. (2018)
GPR39	Promoting keratinocyte proliferation, migration, and barrier repair	Activation of MAPK/ERK and PI3K/AKT signaling pathways, with Zn^2+^ and TC-G 1008 as key agonists	Zn^2+^ TC-G 1008(Agonist)	Hershfinkel (2018), Laitakari et al. (2021), Chatkul et al. (2025)
β1/β2-AR	Modulates​ fibroblast activity to reduce excessive collagen deposition during the remodeling phase	Functions as a non-selective beta-adrenergic receptor antagonist	Timolol (antagonist)	Hawwas et al. (2023); Nafissi et al. (2025)
BLT2	Promotes keratinocyte migration, enhances re-epithelialization, and improves granulation tissue formation	Upregulates wound healing mediators (e.g., TNF-α, MMP9, TGF-β1, bFGF, nitric oxide) and stabilizes tight junctions via NF-κB signaling	12-HHT, CAY10583 (agonist)	Liu et al. (2014); Luo et al. (2017), Leguina-Ruzzi et al. (2019), Yasukawa et al. (2020)
GPR124	Promotes pericyte migration and polarization during ischemic injury; enhances CNS angiogenesis and barrier integrity	Acts as a ligand-specific co-activator for Wnt7a/Wnt7b canonical signaling; interacts with Reck and vinculin; regulates Cdc42 and filopodia formation	—	Cho et al. (2017), Abadir et al. (2018), Chen et al. (2019), Chatkul et al. (2025), Cui et al. (2026)
CB2	Promotes wound healing by suppressing inflammation, accelerating re-epithelialization, and attenuating fibrogenesis	Restrains M1 macrophage polarization and cytokine release, while also engaging ancillary pathways (e.g., Nrf2/HO-1, TRP channels) to resolve inflammation and promote cell migration	JWH-133, GP1a, Beta-caryophyllene (agonist)	Karsak et al. (2007), Wang et al. (2016), Koyama et al. (2019), Wu et al. (2020)
CaSR	Promots keratinocyte proliferation, migration, and differentiation; facilitating collective re-epithelialization and epidermal barrier repair	Activates Gαq/11-PLC-IP3 pathway leading to Ca2+ release; stabilizes E-cadherin/catenin complexes to engage EGFR/MAPK/ERK and PI3K/Akt signaling; modulates SOCE via STIM1	NPS R-568, (agonist)	Tu et al. (2019), Celli et al. (2021)
IP receptor	Accelerates​ the healing of ischemic and diabetic ulcers by improving tissue perfusion, reducing inflammation, and inhibiting platelet aggregation	Activates​ the cAMP/PKA signaling pathway to induce vasodilation and cytoprotective effects	Beraprost sodium (agonist); Iloprost (agonist); Treprostinil (agonist)	Berman et al. (2006), Awsakulsutthi et al. (2014), Kotzki et al. (2022), Legrand et al., 2025
HRH1	Reduces inflammation and itch	Antagonizes H1 receptor to inhibit histamine-induced pruritus and vascular permeability	Diphenhydramine (antagonist)	Shenefelt (2010); Marsella and De Benedetto (2017); De Nardi et al. (2022)
ETA/ETB	Accelerates healing of refractory lower extremity ulcers; prevents new ulcer formation; promotes granulation tissue formation and re-epithelialization	Antagonizes ET-1 receptors to reduce vasoconstriction and improve perfusion, alleviating microangiopathy-related ischemia	Bosentan (antagonist)	Naert and De Haes (2013); Huh et al. (2021)

Despite this promise, clinical translation remains constrained by pronounced context dependence and unresolved biological uncertainties. Individual GPCRs may exert opposing effects across different phases of repair or tissue environments; for example, PAR1 promotes early fibroblast migration and wound closure, whereas sustained activation drives pathological fibrosis (Xie et al., 2016). Similarly, β-adrenergic receptor agonists inhibit keratinocyte migration in skin and corneal epithelia but elicit divergent responses in other epithelial tissues, such as the airway, complicating systemic administration (Cancado et al., 2015; Johnston et al., 2026). Progress is further limited by the persistence of orphan GPCRs, with over 100 receptors still lack identified endogenous ligands (Ahmad et al., 2025). The heavy reliance on immortalized cell lines with artificial receptor overexpression also limits the translational relevance of many preclinical findings. Consequently, despite the apparent druggability of GPCRs, over 90% of candidates fail to advance past Phase I clinical trials (Mishin et al., 2019). Nevertheless, GPCR-targeted intervention remains a promising strategy for enhancing wound repair, provided that therapeutic development is guided by improved mechanistic resolution. Given the high conservation of orthosteric sites among GPCRs and the associated risk of off-target effects, allosteric modulation offers an attractive alternative by enabling greater receptor selectivity. Continued deorphanization efforts and the discovery of novel biased agonists or antagonists are expected to expand the functional repertoire of GPCR signaling and open new avenues for the development of safer and more effective wound-healing therapies.
